# Research progress on fecal microbiota transplantation in tumor prevention and treatment

**DOI:** 10.1515/biol-2022-0954

**Published:** 2025-04-01

**Authors:** Yijia Wu, Xi Chen, Qingming Wu, Qiang Wang

**Affiliations:** Wuchang Hospital Affiliated to Wuhan University of Science and Technology, Medical College, Wuhan University of Science and Technology, Wuhan, 430065, China

**Keywords:** gut microbiota, dysbiosis, cancer, fecal microbiota transplantation, therapy

## Abstract

The application of fecal microbiota transplantation (FMT) as a therapeutic strategy to directly modify the makeup of the gut microbiota has made significant progress in the last few decades. The gut microbiota, a sizable microbial community present in the human gut, is essential for digestion, immunomodulation, and nutrition absorption. Alternatively, a growing body of research indicates that gut microbiota is a key contributor to cancer, and intratumoral bacteria are considered to be crucial “accomplices” in the development and metastasis of malignancies. The exceptional clinical effectiveness of FMT in treating melanoma patients has been adequately established in earlier research, which has created new avenues for the diagnosis and treatment of cancer and sparked an increasing interest in the treatment and prevention of other cancers. However, further research on the function and mechanisms of the gut microbiota is required to properly comprehend the impact and role of these organisms in tumor regulation. In this article, we present a detailed account of the influence of FMT on the entire course of cancer patients’ illness and treatment, from tumor development, metastasis, and invasion, to the impact and application of treatment and prognosis, as well as address the associated mechanisms.

## 1 Gut microbiota and fecal microbiota transplantation (FMT)

### 1.1 Gut microbiota

Dozens of phyla of bacteria have been identified in the gut microbiota, including *Bacteroidetes*, *Firmicutes*, *Proteobacteria*, *Actinobacteria*, *Verrucomicrobia*, *Fusobacteria*, *Cyanobacteria*, and *Spirochetes*. Among these, *Bacteroidetes*, *Firmicutes*, *Proteobacteria*, and *Actinobacteria* are dominant, occupying approximately 99% of the human intestinal bacterial population. Gut microbiota can be divided into three categories according to their biological functions: beneficial bacteria, including *Bifidobacterium*, *Lactobacillus acidophilus*, *Lactobacillus*, etc.; conditionally pathogenic bacteria, including *Enterococcus*, *Escherichia coli*, *Proteus bacillus*, etc.; and harmful bacteria, including *Salmonella*, *Staphylococcus*, and *Pseudomonas aeruginosa*, etc.

### 1.2 Influence of gut microbiota in human diseases

In recent years, there has been a growing discovery that the gut microbiota plays an important role not only in gastrointestinal diseases but also in non-intestinal diseases. Based on the theoretical basis that gut microbes can alter the composition of the gut microbiota and influence the release of gut hormones, gut microbes may provide a powerful therapeutic target for the treatment of metabolic diseases and obesity symptoms [1]. There is evidence that a bidirectional gut-brain communication link exists between the central nervous system (CNS) and gut bacteria, and that ecological dysregulation of the gut microbiota is associated with a number of diseases affecting the CNS [2]. Besides, gut-derived metabolites have also been associated with several cardiovascular diseases (CVD). Trimethylamine N-oxide and phenylacetylglutamine (PAG) have been shown to be gut flora-dependent metabolites causally associated with CVD [3]. In addition, abuse of alcohol leads to dysbiosis of the gut microbiota, which worsens liver injury and damages the intestinal barrier [4]. Gastritis and maybe gastric cancer in the host can result from *Helicobacter pylori* infection colonization of the gastrointestinal tract [5]. Dysbiosis of gut microbiota in non-obese diabetic mice induces the production of type I interferon by colonic epithelial cells, which ultimately promotes pancreatic autoimmune antisense and the development of type I diabetes [6] (Table 1). There is growing evidence that gut microbiota plays a significant role in both gastrointestinal and non-intestinal disorders, and there is a close relationship between several diseases. The development of gut microbiota research in tumor prevention and treatment is comprehensively summarized in this study.

**Table 1:** Influence of gut microbiota in human diseases

Diseases category	Gut microbiota	Functions and connections	References
Neurological disorders (e.g., Alzheimer’s disease, autism spectrum disorder, multiple sclerosis, Parkinson’s disease)	Intestinal dysbiosis	Gut–brain axis: immune pathway: cytokines; microbial metabolites; the neuroactive pathway: neuroactive metabolites and neurotransmitters; the neural pathway: spinal nerves, enteric nervous system, and vagus nerve; the endocrine pathway; the hypothalamic–pituitary–adrenal axis	[2]
CVD (e.g., atherosclerosis, thrombosis, and vascular inflammation)	Imbalance of intestinal microbiota composition	Gut microbiota-dependent metabolites: trimethylamine N-oxide and PAG are associated with incident CVD risks; PAG promotes adverse cardiovascular phenotypes via interaction with multiple adrenergic receptors	[3]
Hepatic disorders (e.g., alcohol-induced liver disease, non-alcoholic fatty liver disease [NAFLD], non-alcoholic steatohepatitis [NASH], drug-induced liver injury)	Disruptions in intestinal barrier; Increased intestinal permeability; patients with NAFLD or NASH have less abundance of butyrate-producing bacteria	Disruptions in the intestinal barrier can result in the translocation of microbes into the blood and sustained inflammatory response that promotes liver injury, fibrosis, cirrhosis, and oncogenic transformation. Intestinal barrier dysfunction and dysbiosis contribute to the development of diseases in the liver and other organs	[4]
Gastrointestinal diseases (e.g., gastritis, gastric malignancy, CRC)	Colonization of pathogenic bacteria	*H. pylori* promote tumorigenesis via the activation of β-catenin signaling pathway; diagnostic bacterial markers; CRC-enriched bacteria: *Bacteroides fragilis, F. nucleatum, Porphyromonas asaccharolytica, Parvimonas micra, Prevotella intermedia, Alistipes finegoldii*, and *Thermanaerovibrio acidaminovorans*	[5]
Diabetes (e.g., autoimmune type 1 diabetes)	Defective cathelicidin-related antimicrobial peptide (CRAMP) expression inducing dysbiosis	CRAMP expression promotes the pancreatic autoimmune response in adults and the development of diabetes	[6]

### 1.3 Reconstruction of gut microbiota through FMT

FMT involves introducing microorganisms from a healthy donor’s feces into a patient’s gut to treat illnesses linked to dysbiosis. The functional flora in the feces is what is transplanted, not the feces themselves, in order to restore the biological function and diversity of the patient’s gut microbiota. It has been reported that immunoglobulin A (IgA) constitutes the most prevalent antibody present on the mucosal surface, attributed to its unique structural features and secretory composition, which enable its survival on the mucosal surface [7]. FMT was found to largely reshape the IgA–flora interaction pattern in the intestine of patients with recurrent *Clostridium difficile* infection (RCDI) toward healthy fecal donors [8]. The predominant features of the gut microbiota of RCDI patients were a rise in the percentage of *Proteobacteria*, a notable drop in *Firmicutes*, and the removal of *Bacteroidetes*. The proportion of the patient’s gut microbiota that matched the donor’s was very close 24 h after FMT was carried out, reflecting the significant effect of FMT treatment in the short term [9].

## 2 FMT and tumor development

### 2.1 Imbalance of gut microbiota and tumorigenesis

The gut microbiota has a profound effect on the homeostasis of the intestinal immune system, and components of the microbiota have been shown to both trigger inflammation and modulate immune cells within the gut lamina propria [10]. Dysregulation of the gut microbiota has a substantial impact on the development of colorectal cancer (CRC), with *Fusobacterium*, *Bacteroides*, *Parvimonas*, and *Prevotella* being widely distributed throughout the CRC tumor microenvironment [11]. Besides, the oral cavity and colon, although distant from the anatomical region, are both highly colonized by different microbiota [12]. However, studies have shown that oral bacteria are able to spread into the colon. This is most evident in conditions such as periodontitis, where specific bacteria, *Fusobacterium nucleatum* and *Porphyromonas gingivalis*, have pathogenic characteristics. Within the intricate biofilms of the colon, these bacteria have the potential to modify the composition of the residual microbiota, thereby inducing intestinal dysbiosis and promoting aberrant immune and inflammatory responses. These aberrant reactions ultimately contribute to the pathogenesis of CRC tumorigenesis [13]. In addition, *F. nucleatum* induces a pro-inflammatory microenvironment by attracting tumor-infiltrating immune cells, inhibits immune attack by binding its bacterial protein Fap2 to the inhibitory immune receptor TIGIT on natural killer cells and T cells, and promotes E-cadherin/β-catenin signaling via FadA adhesin [14]. Understanding the exact mechanisms of these interactions will lead to new opportunities for the prevention and treatment of CRC (Table 2).

**Table 2:** Functions of FMT

Disease	Microbiota characteristics	Symptomatic behavior	FMT-associated microbiota changes	Influence and effect	Mechanism
Tumorigenesis (e.g., 1)	Dysbiosis of gut microbiota with high colonization by pathogenic bacteria (e.g., *Fusobacterium*, *Bacteroides*, *Parvimonas,* and *Prevotella*)	Dysregulation of the gut microbiota; pathogenic bacteria (e.g., *F. nucleatum*) trigger the inflammatory microenvironment; and abnormal immune response	FMT-mediated restoration of gut microbiota diversity and IL-9 levels; suppression of ecological dysregulation of the host microbiota and tumor susceptibility due to long-term injectable antibiotic therapy	FMT leads to tumor regression and restoration of gut microbiota levels	Competition with indigenous gut microbiota; promote anti-inflammatory factor expression; restoration of anti-cancer immune cell frequency; tryptophan metabolites reduce stress adaptation in tumor cells; and increased expression of homeostatic genes and repressed inflammatory genes
Tumor metastasis (e.g., breast cancer, thyroid cancer; clear cell renal cell carcinoma)	A high abundance of *F. nucleatum*, *Hungatella hathewayi, Proteobacteria,* and *Sphingomonas* was found to be closely associated with tumor metastasis; *Streptococcus lutetiensis* can promote cancer cell proliferation, migration, and invasion	Tumor-resident intracellular microbiota (e.g., *Enterococcus, Streptococcus*) promotes metastatic colonization in breast cancer; intracellular bacteria enhance the viability of tumor cells under mechanical stress	Probiotics, especially *Lactobacillus* and *Bifidobacterium* species, and their metabolites (e.g., propionate or butyrate) function systemically by increasing the number/activity of immune cells, and that effector T cells infiltrated into tumors; increase in beneficial bacteria *Lachnospiraceae*, *Streptococcus*, and *Lachnoclostridium* involved in the production of SCFAs	Elimination of tumor-resident microbes impedes metastasis	Gut microbiota regulates cancer metastasis in a circRNA/miRNA-dependent manner; restoration of intestinal commensal bacterial diversity and community richness; probiotic mixture enhances helper T-cell recruitment to metastatic tissues and attenuates cancer cell metastasis; metabolites propionate and butyrate inhibit tumor metastasis
Refractory RCDI	Complete disappearance of *Bacteroidetes*, a marked reduction in *Firmicutes,* and high levels of *Proteobacteria*	Loss of microbial diversity	Enhanced overall microbial diversity; Restoration of *Bacteroidetes* and *Firmicutes* dominance in fecal bacterial composition and *Proteobacteria* are reduced after FMT treatment	Donor microbiota become established and engrafted into recipients of FMT with refractory RCDI	Competition for nutrients; direct suppression by antimicrobial peptides (e.g., thuricin CD produced by *Bacillus thuringiensis* isolated from the human intestine; nisin produced by *Lactococcus lactis*); bile-acid-mediated inhibition of spore germination and vegetative growth; and activation of immune-mediated colonization resistance
syndrome					


### 2.2 FMT leads to tumor regression and restoration of gut microbiota levels

FMT is reported to be the most innovative approach to gut microbiota modification, defined as the transplantation of a healthy donor’s gut microbiota into a diseased patient via the upper or lower gastrointestinal tract [15]. Studies have shown that IL-9-producing T cells have potent anti-cancer properties [16]. Germ-free (GF) mice have a low frequency of IL-9-producing T cells in the lamina propria of the colon compared to conventional mice, and reconstitution of the gut microbiota restores the cell frequency. Besides, prolonged antibiotic treatment by injection promotes ecological dysregulation of the host microbiota and heightens susceptibility to tumor formation, while FMT replenishes the diversity of the gut microbiota and IL-9 levels, ultimately suppressing tumorigenesis [17]. Not only that, but the impact of fecal transplants on chemotherapy treatment cannot be ignored. The down-regulation of the reactive oxygen species (ROS)-degrading enzymes glutathione peroxidase 3 and glutathione peroxidase 7 was brought about by the combined effects of chemotherapy and indole-3-acetic acid, a tryptophan metabolite of microbial origin from fecal transplants. The increased accumulation of ROS also hindered the cancer cell’s capacity to adapt to stress, which in turn reduced the proliferation of pancreatic ductal adenocarcinoma (PDAC) cells [18].

## 3 FMT and tumor metastasis

### 3.1 Changes in the intratumoral microbiota promote tumor metastasis

Bacteria are present in highly immunosuppressive microcosms within the CD25+ immune zone in oral squamous cell carcinoma and CRC tumors. Characterized by the enrichment of mature CD66b+ myeloid cells and upregulation of the immunosuppressive molecules arginase 1 and the immune checkpoint protein CTLA4. Notably, the distribution of the intra-tumor microbiota is not random; rather, it is highly organized in microecological niches with immune and epithelial cell functions that promote cancer progression [19]. In addition, abnormal DNA methylation has been reported as one of the characteristics of thyroid cancer (TC) and is associated with the development of TC [20]. By upregulating DNA methyltransferases, methylation-regulating bacteria like *F. nucleatum* and *Hungatella hathewayi*, which are common in CRC, may contribute to the development and spread of TC [21]. A high abundance of *Sphingomonas* was found to be closely associated with tumor metastasis to the lymph nodes, and the abundance and diversity of thyroid flora was lower than that in the tissues surrounding the tumor in TC specimens [22] (Table 2). Moreover, Chen et al. found for the first time substantial variations in the distribution of gut microbiota between patients with clear cell renal cell carcinoma (ccRCC) and healthy controls using a 16S rRNA sequencing study. Five taxa, namely *Blautia*, *Streptococcus*, *Ruminococcus torques* group, *Romboutsia*, and *Eubacterium hallii* group, were found in large quantities in the intestines of patients with ccRCC. By activating the TGF-b signaling system, *Streptococcus lutetiensis* can enhance the migration, invasion, and proliferation of ccRCC cells [23]. In previous studies, it was found that *F. nucleatum* usually reaches tumors via the hematogenous route and specifically attaches to tumors via Fap2–Gal–GalNAc interactions, accelerating breast cancer progression and that lung metastases are more frequent in the presence of *F. nucleatum* [24]. In addition, *F. nucleatum* may have promoted CRC invasion and metastasis by down-regulating the m6A methyltransferase METTL3, which in turn reduced m6A modifications in CRC cells and patient-derived xenografts. *F. nucleatum* up-regulation of KIF26B, a key gene in CRC metastasis, had a similarly promoting effect on CRC development [25]. Another study on tumor metastasis found that *E. coli* enhanced the invasive capabilities of non-small-cell lung cancer cells in persons with lung cancer in a dose-dependent manner, encouraging rapid proliferation and metastasis of lung cancer cells [26].

### 3.2 FMT regulates tumor metastasis

It was found that broad-spectrum antibiotic-mediated depletion of gut microbiota significantly promoted tumor metastasis in GF mice. In contrast, the transplantation of fecal microbiota from specific pathogen-free mice into GF mice significantly suppressed lung metastasis. The specific mechanism is that gut microbiota can regulate cancer metastasis in a circRNA/miRNA-dependent manner [27] (Table 2). In addition, modulation of gut commensal bacterial diversity and community richness may inhibit CRC liver metastasis, as found in a treated group of BALB/c mice with splenic tumor injections leading to CRC liver metastasis. Immunohistochemical assessment of liver tissue Kupffer cell (KC) content in tumor-bearing mice pretransplant with *Proteus mirabilis* or *Bacteroides vulgatus* confirmed that *P. mirabilis* or *B. vulgatus* may potentially affect KC and be effective in the treatment of liver metastases [28]. Chen et al. demonstrated that *Bifidobacterium longum*, *Bifidobacterium breve*, *Lactobacillus infantis*, *L. acidophilus*, *Lactobacillus plantarum*, *Lactobacillus casei*, *Lactobacillus bulgaricus*, and *Streptococcus thermophilus* could attenuate the development of lung metastases in melanoma mice and achieve prolonged survival [29]. Besides, Shang et al. have shown that in a CRC mouse model, the addition of an intestine probiotic mixture of *Bifidobacterium bifidum*, *L. acidophilus*, and *L. plantarum* inhibited the growth and even the incidence of cancer cell metastasis [30].

## 4 FMT and prognosis of tumor patients

### 4.1 Specific gut microbiota can be used as a monitoring factor for poor prognosis and FMT improves long-term survival in tumor patients

**Figure 1:**
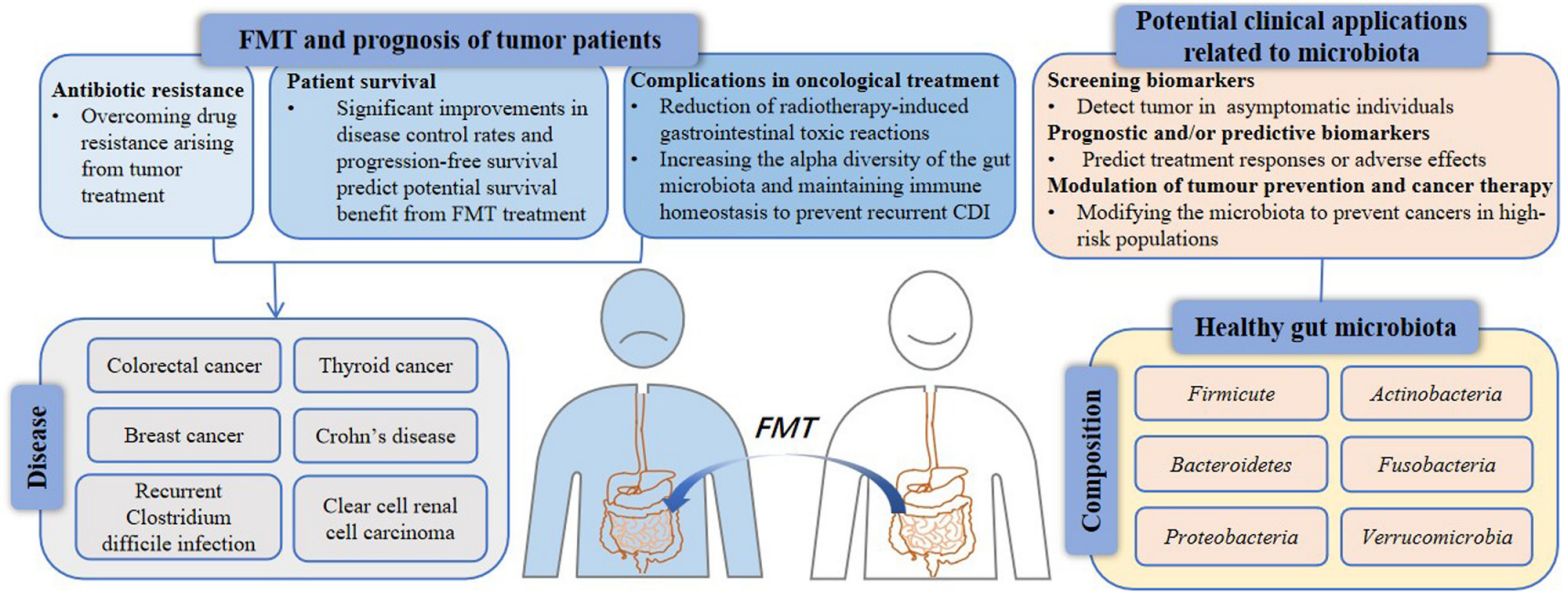
Clinical application values of microbiota and FMT. This article describes several potential clinical applications of FMT for the use of microbiota in tumor prevention and therapy, including prevention of tumorigenesis, modulation of tumor metastasis, reduction of tumor treatment complications, screening, prognostication, and prediction of tumor biomarkers. Emphasis is placed on the role of gut microbiota in human disease. FMT, fecal microbiota transplantation.

Numerous studies have shown that specific gut microbiota may serve as a monitoring factor for poor prognosis. Compared to non-inflammatory bowel disease controls, the gut microbiome of patients with active inflammatory bowel disease (IBD) is altered in membership and function, characterized by an overall decrease in species diversity, a decrease in the *Firmicutes* and an increase in the *Proteobacteria* [31]. Members of the *Firmicutes* and *Bacteroidetes* families dominate the gut microbiome in non-alcoholic fatty liver disease (NAFLD), with substantially less *Proteobacteria* and *Actinobacteria* in second and third place. However, as the disease progresses from mild/moderate NAFLD to advanced fibrosis, there is a statistically significant increase in *Proteobacteria* abundance and a decrease in *Firmicutes* [32]. Research has revealed that patients with pancreatic adenocarcinoma who survive for an extended period have tumor microbiomes with higher alpha diversity. Intra-tumor microbiome profiles have been identified, with *Saccharopolyspora*, *Bacillus clausii*, *Streptomyces*, and *Pseudoalteromonas* being highly predictive of long-term survival [33]. The diversity and composition of the PDAC tumor microbiome can affect immune infiltration and, ultimately, the survival of PDAC patients [34]. Furthermore, patients with advanced gastro-esophageal cancer treated with capecitabine and oxaliplatin or dual chemotherapy had a higher survival rate with allogeneic FMT in a randomized controlled trial (RCT) of FMT in healthy obese donors. The significant improvement in disease control rate and progression-free survival suggests that FMT treatment may benefit patients with advanced gastro-esophageal cancer in terms of survival [35]. In summary, specific gut microbiota can be used as a monitoring factor for poor prognosis and FMT can benefit the survival of oncology patients (Figure 1).

### 4.2 Dysbiosis of gut microbiota can lead to resistance to drug therapy, FMT overcomes resistance arising from oncology treatment

In preclinical models of epithelial ovarian cancer (EOC), it has been established that dysregulation of the gut microbiota in patients with EOC following antibiotic treatment leads to enhanced tumor growth and reduced sensitivity to platinum chemotherapy [36]. Besides, in patients with metastatic CRC, increased levels of *F. nucleatum* and the metabolite succinic acid in the gut inhibit the cGAS-interferon-β pathway, which in turn inhibits the transport of CD8+ T-cells into the tumor microenvironment, leading to patient resistance to immunotherapy [37]. Notably, the human gut microbiota is now considered to be the repository of determinants of antibiotic resistance, known as the “gut resistome.” A number of conditionally pathogenic bacteria that are particularly parasitic in the gut, including *C. difficile*, *E. coli*, and *Enterococcus faecalis*, are widely infected in immunocompromised hosts. The acquisition of mobile genetic elements carrying antibiotic-resistance genes by bacteria through the process of horizontal gene transfer contributes to the emergence of multidrug-resistant clones of opportunistic pathogens [38]. FMT has shown significant potential in restoring the microbiome and reducing the intestinal drug resistance genome. In addition, both *in vivo* and *in vitro* experiments have demonstrated that *Proteobacteria* enrichment in the intestinal microbiota generated by antibiotic treatment can raise intestinal permeability and bacterial lipopolysaccharide levels within the tumor, hence activating the NF-κB-IL6-STAT3 axis. In the end, this strategy encourages mice to develop docetaxel chemoresistance and prostate cancer (PCa) [39]. Melanoma patients have been clinically shown to develop a rapid immune response to anti-PD-1 therapy, and neoadjuvant anti-PD-1 has been effective in the treatment of high-risk resectable III/IV melanomas, but mechanisms of resistance have emerged in patients with relapsed disease [40]. Even with the most effective double-blockade combination of PD-1 and CTLA-4, 50% of patients with advanced melanoma still die due to resistance [41]. However, FMT can change responders’ (Rs) serum metabolome and cytokines. In patients with PD-1-refractory melanoma, circulating biomarkers linked to positive clinical outcomes were upregulated, while several circulating cytokines and chemokines associated with anti-PD-1 resistance were demonstrated to be downregulated in Rs. This ultimately induced a clinical response against PD-1 [42]. Additionally, following FMT in homozygous mice treated with anti-PD-1, the number of intestinal mimics of the genus *Bacillus ovatus* was raised, while the number of *Bacillus fragilis* was decreased. Because of their immunomodulatory properties, elevated levels of metabolites such as aspirin and butyric acid may further enhance the response impact of anti-PD-1 therapy [43]. Not only that, but FMT also enhanced the immune system’s response in Rs. When compared to non-responders, Rs exhibited higher levels of intratumoral CD8+ T cells expressing activation-associated genes, which are useful for immunotherapy, as well as circulating CD8+ CD56+ T cells and activated mucosal-associated invariant T cells [44].

### 4.3 FMT reduces complications in oncology treatment

#### 4.3.1 FMT reduces radiotherapy-induced gastrointestinal toxicities

In previous studies, it was found that FMT may be used as a therapeutic approach to reduce radiotherapy-induced toxicity and improve the prognosis of patients with tumors after radiotherapy (Figure 1). In *in vivo* experiments, FMT increased the survival of irradiated animals, increased peripheral blood leukocyte counts, and improved gastrointestinal function and intestinal epithelial integrity in irradiated male and female mice [45]. Some probiotics producing short-chain fatty acids (SCFAs), especially *Lactobacillus*, *B. bifidum*, *Faecalibacterium prausnitzii*, and *Akkermansia muciniphila*, have been reported to have a positive effect on radiation injury [46]. *Lachnospiraceae* and *Enterococcaceae* were found to be the most abundant taxa in survivor fecal samples and were associated with post-radiation recovery of hematopoietic and gastrointestinal tract repair in a mouse model of radiation injury treatment [47]. Long-term radioprotection *in vivo* is provided by the tryptophan pathway metabolites 1h-indole-3-carboxaldehyde and kynurenine of both groups [48]. Not only that, in a mouse model of acute radiation-induced intestinal damage, the probiotic combination treatment had beneficial effects [49]. To improve stool consistency, *L. acidophilus* and *B. longum* worked together to lessen grade 2–3 diarrhea brought on by radiation enteritis [50]. Besides, FMT was found to increase the levels of microbial-derived indole-3-propionic acid (IPA) in the fecal pellets of irradiated mice, and mice supplemented with IPA by oral route exhibited lower levels of systemic inflammation, restoration of hematopoietic organs, alleviation of bone marrow suppression, and improvement in gastrointestinal function and post-irradiation epithelial integrity [51].

#### 4.3.2 FMT as a therapeutic approach for RCDI induced by chemotherapy

Numerous factors, including advanced age, multimorbidity, immunocompromised state, frequent use of antibiotics and chemotherapy, hematopoietic stem cell transplantation, nasogastric tube feedings, and repeated hospitalization, enhance the incidence of RCDI in cancer patients. Together, these variables elevate the risk of RCDI in cancer patients [52]. *Proteobacteria* levels are high in samples from patients with RCDI, and the relative proportions of *Firmicutes* and *Bacteroidetes* are frequently increased, while *Proteobacteria* levels are decreased after FMT treatment [53] (Table 2). Compared to healthy people and patients experiencing their first episode of *C. difficile* infection (CDI), patients with RCDI statistically have higher feces levels of primary bile acids (BAs), such as taurocholic acid, which promotes *C. difficile* spore germination [54]. Research evidence suggests that the effectiveness of FMT derives not only from the ability of the donor probiotic microbiota to clear *C. difficile* from infection but also that its production of key metabolites, such as BAs and SCFA, reduces the activation of the innate and adaptive immune systems by pathogen-derived antigens. Through this mechanism, FMT promotes the expansion of Treg cells and increases the production of the anti-inflammatory factor IL-10, thereby contributing to the maintenance of intestinal immune homeostasis and promoting disease recovery [54]. Additionally, FMT increased the alpha diversity of the colonic microbiota and the amount of IL-25 in colonic tissue, which prevented recurrent CDI in RCDI patients by inducing the expression of IL-25 in the colon [55].

## 5 Discussion

### 5.1 The significance of FMT in the prognosis of tumor therapy

This article reviews the impact of FMT in the whole process of disease and treatment of tumor patients, from tumor development, and metastatic invasion, to treatment prognosis, and elaborates on the application of FMT in these three segments in points. In recent years, the use of immune checkpoint inhibitors (ICIs) in tumor therapy has gradually emerged but is prone to drug resistance. FMT not only overcomes patients’ drug resistance but also may reduce radiation therapy-related toxicity or enhance ICI efficacy in the face of serious toxicity caused by the synergistic effects of the two treatments, namely radiation therapy and ICI [56,57]. Advances in the study of FMT in overcoming anti-PD-1 resistance in melanoma patients have led many researchers to consider whether resistance could also be improved by FMT in patients with other tumors where ICIs are used [58]. Research studies show that FMT has been found to play a key role in the development of and response to treatment in ICI-resistant urological tumors including uroepithelial carcinoma, RCC, and PCa [59]. Besides, with 70% of bacteremia cases in hematological oncology patients originating from the gut, FMT may be useful as a safe modality to treat patients undergoing allogeneic hematopoietic cell transplantation and suffering from antibiotic-resistant bacteria colonization [60]. In addition, in response to epidemiological trends and poor prognosis, it is projected that PDAC will likely become the second most highly lethal cancer worldwide by 2040 [61]. FMT is expected to reduce PDAC cell proliferation by impairing the cancer cell’s ability to respond to stress, providing a new way of thinking about the clinical diagnosis and treatment of patients with metastatic PDAC [18]. Intratumoral bacteria are important “accomplices” in tumor development and metastasis, and by regulating the gut microbiota, FMT may help to reduce the risk of tumor metastasis [62]. In patients with tumors created by conventional radiation, FMT is anticipated to reduce gastrointestinal toxicity and preserve epithelial integrity. Through immunomodulation and metabolism, the gut microbiota may potentially enhance immunotherapy resistance, which could have a significant favorable impact on long-term patient survival [63]. Additionally, by increasing the abundance of beneficial bacteria – such as *Bacteroidetes*, *Lactobacillus*, and *Prevotella* – that have been diminished as a result of radiation, FMT restores the diversity of the patient’s microbiome and reestablishes the balance of the gut microbiota and is anticipated to be a novel approach to mitigate side effects.

### 5.2 Challenges and benefits of FMT

**Figure 2:**
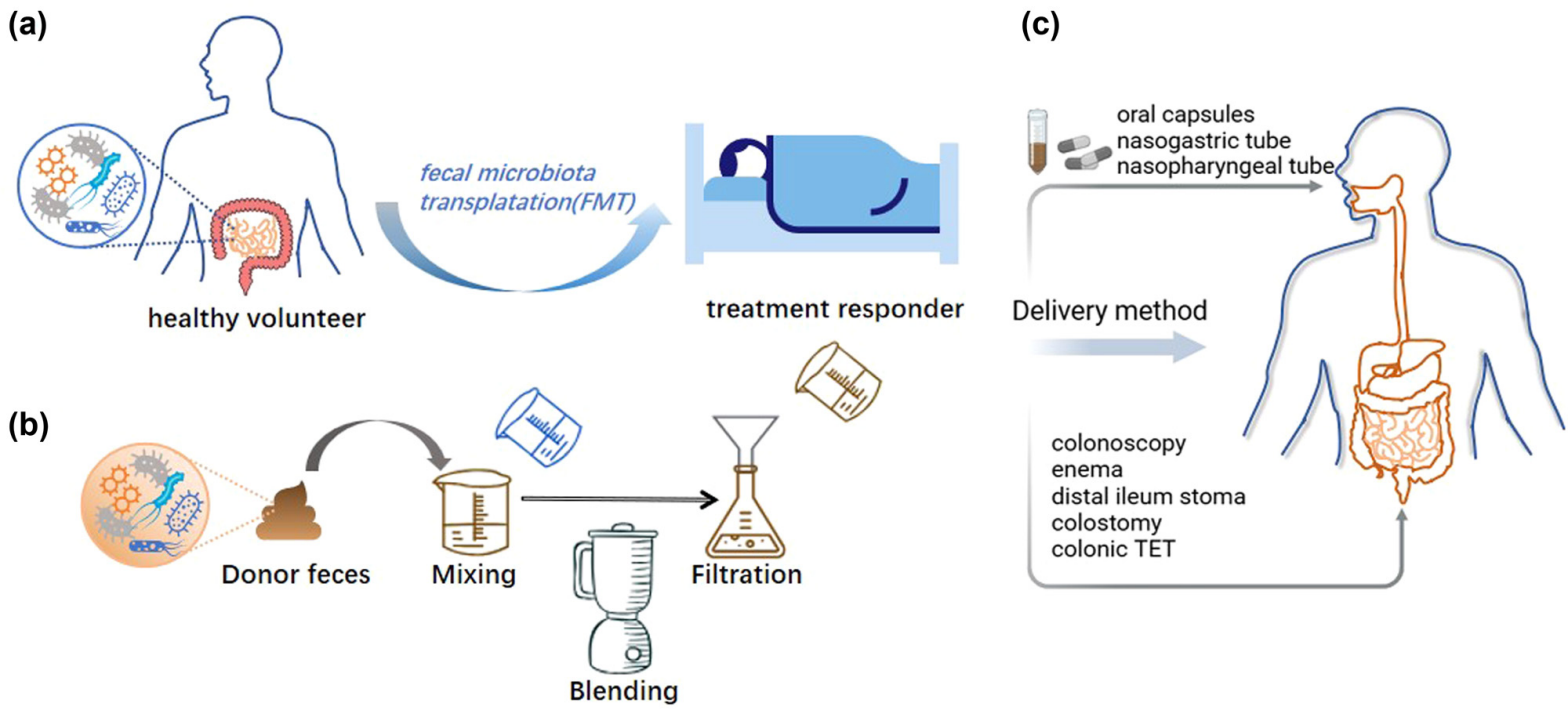
Methodology and regulations of FMT: (a) donor selection and transplantation; (b) manual preparation of fecal microbiota; and (c) delivery of FMT.

Notably, it is impossible to overlook FMT’s limits in the treatment of cancer. Certain anticancer medications (such as gemcitabine and irinotecan) can become ineffective or have more side effects due to microbial metabolism [64]. In terms of donor selection, the US Food and Drug Administration has recommended that FMT donor screening must include a fecal testing questionnaire specifically addressing risk factors for colonization by multidrug-resistant organisms (MDROs) and MDROs [65]. Screening for the most effective donor and matching it to a suitable recipient, i.e., the donor–recipient matching principle, is considered to be the key to improving the success of FMT. So far, a set of blood and fecal tests has been proposed to ensure the safety of the FMT procedure. This new strategy narrows the perceived differences in donor screening protocols at different FMT centers [66]. Furthermore, more RCTs with bigger sample sizes are required to evaluate the efficacy of FMT in treating persistent low-grade inflammation. The use of colonic trans-endoscopic enterostomy tubes for microbiota administration, washing microbiota transplantation (WMT), and purifying *Firmicutes* spores from feces are examples of how FMT medicinal techniques and technologies are progressing. The development history of gut microbiota transplantation includes the advancement of transplantation technology and the expansion of indications, from fresh gut microbiota transplantation to standard frozen gut microbiota transplantation to WMT, becoming increasingly personalized and even synthetic microbial therapy. WMT as a new and improved method of FMT makes the delivery of precise doses of microbiota more possible [67]. FMT can also be delivered into the bowel by colonoscopy, enema, distal ileostomy, colostomy, and colonic TET (Figure 2). It has been found that of all delivery methods, colonic TET may be the safest, with the lowest incidence of delivery-related adverse events [68].

Compared to FMT, prebiotics and postbiotics act as health regulators of the symbiotic microbiota, enhancing flora metabolism and demonstrating antioxidant, anti-inflammatory, and anticancer potential. Synthetic probiotics combine the advantages of both to enhance health benefits and to reduce the risk of infection. The newly proposed concept of probiotic-derived molecules provides research avenues for non-living bacterial therapies and significantly reduces potential infections. FMT, conversely, directly transplants healthy flora and rapidly restores the diversity of flora, which is highly effective and promising for the personalized treatment of flora imbalance diseases. The specific mechanism of action of postbiotic bacteria needs to be explored in depth to optimize the application strategy, and together they will bring new hope to the field of intestinal health [69]. In summary, the above hopes and challenges posed by FMT need to be explored and better solutions proposed by scholars together in the future.

### 5.3 Future perspectives

An increasing amount of research has demonstrated the rationale behind treating Crohn’s disease, ulcerative colitis, and CDI by focusing on the gut microbiota. Additionally, a growing amount of information supports the safety and effectiveness of FMT in patients who also have IBD. All these beneficial aspects lay the foundation for the use of microorganisms in adjuvant tumor therapy. Theoretically, the treatment of FMT should be similar across individuals of different ages, as it centers on the transplantation of gut flora. However, there may be differences in the composition of the gut microbiota in different age groups, which may affect the efficacy of FMT, but there is currently no clear evidence to suggest the need for a significant change in the treatment of FMT due to age. Although there are differences in the composition of the gut microbiota between males and females, the need to change treatments because of gender has not been identified in the available FMT studies. Whether these differences are sufficient to influence treatment outcomes for FMT requires further study. In addition, the influence of socioeconomic status on the treatment approach for FMT may be mainly in the choice of donor and the patient’s lifestyle. Individuals with higher status may have easier access to high-quality donors and better medical care, but this does not directly affect the treatment approach for FMT *per se*. The use of donor FMT has the potential to restore gut microbial function, resulting in illness remission and even reversal in people, according to the clinical evidence now available. The gut microbiota has a major influence on the development of cancer and presents new opportunities for cancer detection and therapy. From a strategic standpoint, FMT is the most straightforward method for modifying the gut microbiota’s makeup. Given the speed at which gut microbiology is developing, FMT has the potential to become a viable cancer therapy option in the near future.
